# Open dataset of theory of mind reasoning in early to middle childhood

**DOI:** 10.1016/j.dib.2023.109905

**Published:** 2023-12-02

**Authors:** Koraima Sotomayor-Enriquez, Hyowon Gweon, Rebecca Saxe, Hilary Richardson

**Affiliations:** a School of Health in Social Science, University of Edinburgh, Teviot Place, Edinburgh EH8 9AG, United Kingdom; b Department of Psychology, Stanford University, 450 Jane Stanford Way, Bldg. 420-280, Stanford, CA 94305, USA; c Department of Brain and Cognitive Sciences, Massachusetts Institute of Technology, 43 Vassar Street, Cambridge, MA 02139, USA; d School of Philosophy, Psychology, and Language Sciences, University of Edinburgh, 7 George Square, Edinburgh EH8 9JZ, United Kingdom

**Keywords:** Social cognition, False belief, Development

## Abstract

Theory of mind (ToM) reasoning refers to the process by which we reason about the mental states (beliefs, desires, emotions) of others. Here, we describe an open dataset of responses from children who completed a story booklet task for assessing ToM reasoning (*n* = 321 3–12-year-old children, including 64 (neurotypical) children assessed longitudinally and 68 autistic children). Children completed one of two versions of the story booklet task (Booklet 1 or 2). Both versions include two-alternative forced choice and free response questions that tap ToM concepts ranging in difficulty from reasoning about desires and beliefs to reasoning about moral blameworthiness and mistaken referents. Booklet 2 additionally includes items that assess understanding of sarcasm, lies, and second-order belief-desire reasoning. Compared to other ToM tasks, the booklet task provides relatively dense sampling of ToM reasoning within each child (Booklet 1: 41 items; Booklet 2: 65 items). Experimental sessions were video recorded and data were coded offline; the open dataset consists of children's accuracy (binary) on each item and, for many children (*n* = 171), transcriptions of free responses. The dataset also includes children's scores on standardized tests of receptive language and non-verbal IQ, as well as other demographic information. As such, this dataset is a valuable resource for investigating the development of ToM reasoning in early and middle childhood.

Specifications Table

**Table:** 

Subject	Developmental and Educational Psychology
Specific subject area	Children's “theory of mind” reasoning: the process by which they reason about the mental states (beliefs, desires, emotions) of others.
Data format	Analyzed
Type of data	Spreadsheet
Data collection	Participants were 321 3–12-year-old children. Data were acquired via in-person administration of our theory of mind (ToM) booklet task, over the course of eight years (2009–2017). The task involves an experimenter telling a scripted story and asking two-alternative forced choice and free response questions that evoked reasoning about the characters’ mental states. Experimental sessions were video recorded; data were subsequently coded and transcribed offline by trained researchers. ToM booklet task materials, example administration videos, and guidelines for coding are publicly available alongside the dataset (https://osf.io/g5zpv/).
Data source location	Institution: Massachusetts Institute of Technology City/Town/Region: Cambridge, MA Country: USA
Data accessibility	Repository name: Open Science Framework (OSF) Data identification number: 10.17605/OSF.IO/G5ZPV Direct URL to data: https://osf.io/g5zpv/
Related research article	[1] Richardson, H., Riobueno-Naylor, A., Lisandrelli, G., Saxe, R. Development of the social brain from age three to twelve years. Nature Communications, 9(1), 1027. https://doi.org/10.1038/s41467–018–03399–2

## 1 Value of the Data


 

• Theory of mind (ToM) reasoning describes how we reason about the mental states (beliefs, desires, emotions) of others. Key questions about ToM reasoning remain open: there are ongoing debates about developmental milestones in ToM (e.g., false belief reasoning [2,3]) and about the cohesiveness of ToM as a construct [4]. To allow further investigations into these debates, and to improve transparency and reproducibility in ToM research, we generated a dataset of 321 3–12-year-old children who completed at least one of two versions of a ToM story booklet task.
• Most ToM tasks consist of a small number of items and focus on preschool-aged children's understanding of desire, knowledge, and belief. Furthermore, open datasets of children's ToM reasoning are scarce (https://osf.io/g5zpv/), especially those that provide item-level responses beyond composite scores. Our task includes a large number of items (Booklet 1: 41 items; Booklet 2: 65 items), including two-alternative forced choice and free response items. Our dataset provides accuracy for individual items as well as transcripts of free responses. Because our task consists of many items tapping a range of ToM constructs, the dataset provides a rich window into ToM development.
• Participants are 321 3–12-year-old children, including 64 (neurotypical) children tested longitudinally and 68 autistic children. The relatively large amount of data – per participant and the overall sample size – enables fine-grained analyses of different aspects of ToM development within individual children and across a wide age range.
• This dataset is of particular interest to researchers who study the development of ToM reasoning and social cognitive development more broadly, across a range of disciplines (e.g., developmental psychologists, cognitive neuroscientists, clinical researchers, education researchers). Exploration of these data may help researchers generate new hypotheses about ToM development in neurotypical and autistic children or offer preliminary results that motivate further research. For example, this dataset can be used to investigate differences in ToM performance by age, question format, and/or concept.
• Given the dataset includes neurotypical and autistic children, and includes scores from other cognitive assessments (e.g., receptive language, non-verbal IQ) in addition to ToM, this dataset will be of interest to researchers studying autism spectrum disorder and/or children's language. For example, this dataset could be used to investigate differences in ToM across neurotypical and autistic children and/or developmental differences in children's production of mental state language.
• More generally, this dataset can be used to gain further insights into open debates concerning the nature and timing of developmental milestones in ToM (e.g., false belief reasoning) and about the conceptual cohesiveness of ToM as a construct. Exploration of this rich dataset will aid researchers in the generation of new hypotheses about ToM development in neurotypical and autistic children.

## 2 Data Description

The data have been organized into two spreadsheets, one per booklet task, located in the “Open Data” folder of the Open Science Framework (OSF) webpage (https://osf.io/g5zpv/). Both spreadsheets contain the following tabs: Data, Questions, Explanations, Sub Info, and Data Dictionary. We describe each tab in detail below.

### 2.1 Data

The “Data” tab consists of a row per booklet item per participant. The first four columns of the spreadsheet contain the (A) project (i.e., which study a participant was recruited into), (B) subject identifier, (C) experimenter identifier (i.e., who administered the task), and (D) coder identifier (i.e., who coded and transcribed the data) identifiers, respectively. The next set of columns contains information about the booklet items: (E) page and (F) item number, (G) question type (two-alternative forced choice, explanation, or control), (H) ToM concept subcategory (e.g., common desires, diverse desires, diverse beliefs, false beliefs), (I) ToM concept (e.g. desires, beliefs), (J) question identifier (i.e., a unique code that enables matching questions in the dataset to questions in the task script), (K) the question (e.g., where will Diana look for her snack?). Booklet 1 items were asked in the context of two stories: one about children finding books in a classroom and one about a family's visit to the park. For the Booklet 1 spreadsheet, there is therefore an additional column describing the story that each item was a part of (Books/Parks). Projects that used Booklet 2 had small discrepancies in items included; for the Booklet 2 spreadsheet, there is an additional column containing notes about whether the item was administered in all projects/children.

The final six columns contain (M) whether a child's response was correct (1: yes, 0: no), (N) the transcription of the child's response (used for coding explanation items only), and three binary columns indicating (O) whether the child needed clarification (e.g., asked a question / for the item to be repeated (1: yes, 0: no), (P) whether the item should be included in analyses (1: yes; 0: no [for non-experimenter error related reasons, e.g., child was distracted]), and (Q) whether an experimenter error occurred (1: yes, 0: no). The final column (R) contains any notes, which typically describe experimenter errors or other anomalies in testing protocol (e.g., experimenter switched the order of two questions).

### 2.2 Questions

The “Questions” tab contains an ordered list of the items in the booklet task, alongside their question identifier and notes about whether they were included in all or a subset of projects.

### 2.3 Explanations

The “Explanations” tab hosts a pivot table that enables viewing all explanations for all items, organized by whether the explanation was coded as a correct (1) or incorrect (0) response.

Unlike 2AFC and control questions, evaluating children's free responses as correct or incorrect requires predefined evaluation criteria. Consider a relatively simple “false belief” item: Children are told that earlier in the day, Diana places her snack in the drawer. Then, while Diana is away, someone moves her snack to the desk. Children are then asked, “Where will Diana look for her snack?” Regardless of their response to this question, the experimenter then shows Diana looking in the drawer for her snack and says, “Diana is looking in the drawer for her snack. Why does Diana look in the drawer?” In response to this question, many children say “Because that's where she put it.” In this dataset, we consider this explanation to be a correct response [5,6].

However, this explanation does not explicitly refer to Diana's mental state and does not necessarily indicate that children are reasoning about Diana's beliefs (see [2] for further discussion of this exact response). A different concern is that children may use mental state terms without genuinely comprehending them; that is: free responses could overestimate children's ToM reasoning [2]. Despite challenges in interpreting and coding free responses, performance on explanation items correlates with performance on independent 2AFC items, controlling for age (all bs > 0.26, ts > 2.5, ps < 0.02; see https://osf.io/g5zpv/), suggesting that these items measure ToM reasoning. When possible, we provide transcriptions of the children's responses in addition to binary accuracy. Transcriptions are available for 171 children (Booklet 1: 74/258 children (5–10-year-old neurotypical children, M(SD) age =  6.7(1.5) years), Booklet 2: all 127 children; including 30 neurotypical children assessed longitudinally); free responses to explanation items for the remaining children were not transcribed (due to different priorities and interests across studies that contribute to the dataset). Transcriptions enable researchers to use different custom criteria for evaluating the accuracy of children's free responses and conduct more fine-grained analyses of their content (e.g., children's use of particular mental state words).

### 2.4 Participant Info

The “Participant Info” tab contains information about the project that a participant completed, (if applicable) their subject ID for longitudinal data (which enables pairing data across Booklet 1 and 2 spreadsheets), their date of participation, date of birth, age (in years), gender, autism diagnosis status, race, and handedness. For Booklet 1, a “Transcriptions” column indicates whether free responses to explanation items were transcribed for each participant. This tab also contains parent-responses to questions about the languages their child speaks, number of siblings, autism diagnoses in the family, child diagnosis of ADD/ADHD, medications, diet, and school grade and placement (i.e., private vs. public vs. home-schooled). The final columns contain raw and standardized scores on tests of non-verbal IQ (Kaufman Brief Intelligence Test [KBIT-2] [7]), receptive grammar (TROG [8]), and receptive vocabulary (PPVT-4 [9]). For three- and four-year-old children, we provide raw, scaled, and percentile scores for subtests of the Wechsler Preschool and Primary Scale of Intelligence (WPPSI-IV [10]): block design (non-verbal IQ), receptive vocabulary (verbal IQ), matrix reasoning (non-verbal IQ). For these children we also provide summary scores on the Dimensional Change Card Sort task, which is a measure of executive functions (in particular, flexible rule use in the face of interference - i.e., set shifting and response inhibition [11]). The scores include a Pre/Post switch score (i.e., number of pictures sorted correctly pre- and post- switching the sorting rule), a border score (i.e., number of pictures sorted correctly when the sorting rule depended on the presence of a border surrounding the picture), and a summary score; further details about these scores are provided in Zelazo (2006) [11].

### 2.5 Data Dictionary

The “Data Dictionary” tab provides brief descriptions of the columns in the Data and Sub Info tabs, for ease of use.

## 3 Experimental Design, Materials and Methods

### 3.1 Participants

Participants were 258 5–12-year-old children (M(SD) age = 8.0(1.9) years, 78 girls), including 68 autistic children (M(SD) age = 8.9(1.8) years, 13 girls), who completed Booklet 1 and 127 3–12-year-old children (M(SD) age = 7.6(2.7) years, 62 girls) who completed Booklet 2. A subset of neurotypical children completed both booklets, longitudinally (*n* = 64, Visit/Booklet 1: M(SD) age = 6.8(1.6) years; Visit/Booklet 2: M(SD) age = 8.5(2.1) years, 24 girls). As such, the dataset includes 321 distinct children who completed at least one ToM booklet. All children were native speakers of English with normal or corrected-to-normal vision.

Criteria for autistic children (*n* = 68) included both a clinical diagnosis of autism, Asperger's, or PDD-NOS (DSM-IV) by a specialist in neurodevelopmental disorders and a classification of ‘autism’ or ‘autism spectrum disorder’ on the Autism Diagnostic Observation Schedule (ADOS [12]) conducted by a research-reliable administrator.

All children were recruited from the New England area; many were from the local community (Boston, MA, USA). Neurotypical children were recruited using local parenting listservs, promotional activities, and flyers at libraries and museums. Autistic children were recruited using existing clinical databases (Simons Simplex Collection, SFARI, Autism Consortium). Children were recruited for different neuroimaging studies that all involved completed the ToM booklet task. Data were collected between 2009 and 2016 (Booklet 1) and 2012 and 2017 (Booklet 2).

### 3.2 Task Design and Administration

The ToM booklet task was designed to feel like a naturalistic story telling experience. An experimenter told participants a story from memory while using a binder of illustrations as visual aids. The story described characters in a classroom at snack time. Movable magnetic pieces were used to prompt responses (e.g., “Where will Diana look for her snack” [experimenter hands Diana to the child]) and to maintain attention to the story.

Booklet 1 was developed first and comprises 41 questions (25 two-alternative forced choice [2AFC], 14 explanation, and 2 control). The ToM concepts tested were drawn from research describing the successive ToM achievements in early and middle childhood [13]: common and diverse desires, true and false beliefs [14], and emotions, with the addition of items concerning misleading references [15] and moral blameworthiness [16]. Booklet 2 was developed for longitudinal assessment of ToM reasoning and includes 65 questions (27 2AFC, 25 explanation, and 13 control; note, for one project 1 additional control item was asked). ToM concepts include all of those assessed in Booklet 1 as well as “harder” ToM concepts: second-order belief-desire reasoning, sarcasm [17], and lies [18]. Items that test the same ToM concepts across Booklets 1 and 2 were designed to be directly comparable via longitudinal analyses and therefore are analogous in structure. For 3–4-year-old children, we administered a truncated version of Booklet 2 that omits the most challenging questions, due to time constraints inherent to testing young children and experimenter-perceived difficulty; 3–4 year olds were also administered one “implicit response” item where children responded by placing a character on the page (50 questions total; 20 2AFC (including 1 implicit response item), 20 explanation, 10 control; see https://osf.io/g5zpv/ for details).

For both booklets, the concept categories assigned to items were experimenter-generated, rather than empirically determined (i.e., through analyses of internal consistency). Similarly, item/concept difficulty as described here is based on prior empirical evidence [16], [17], [18], rather than empirical tests within this dataset.

Both versions of the task take approximately twenty minutes to administer. All task materials (scripts and illustrations, as well as examples of administration and coding guidelines) are publicly available via OSF (https://osf.io/g5zpv/).

Experimental sessions were carried out in a quiet room within the Department of Brain and Cognitive Sciences at MIT.

### 3.3 Task Coding

The ToM behavioral task was coded off-line; a researcher watched a video recording of the experimental session, indicated the correctness of responses to 2AFC, control, and explanation questions, and, for many participants, transcribed responses (Booklet 1: *n* = 74/258, Booklet 2: *n* = 127/127; including 30/64 participants who completed both booklets, longitudinally). As described in the Data description section, transcriptions enable researchers to use their own criteria for evaluating the accuracy of free responses to explanation items.

### 3.4 Summary Scores

Researchers are welcome to summarize performance on this task in different ways, depending on their research question and preferences. In prior manuscripts, we have summarized overall performance on the ToM booklet task by calculating the proportion of 2AFC and explanation questions answered correctly [1,19]; we have also calculated summary scores for subsets of questions (e.g., false belief items [1]). Note that as free responses to explanation items are open-ended, it is difficult to determine chance-level performance (but using 50 % chance levels would be conservative).

### 3.5 Psychometric Evaluation

To evaluate the reliability of the ToM tasks, we calculated Cronbach's α and McDonald's ω_t_. Cronbach's α is a standard measure of reliability that assesses the amount of shared variance among test items; McDonald's ω_t_ further represents variance due to n common factors across test items. Both booklets demonstrate very high or excellent reliability, suggesting that there is a common factor underlying the test items within each booklet (Booklet 1: *α* = 0.92 *ω_t_* = 0.94; Booklet 2: *α* = 0.89, *ω_t_* = 0.96). Researchers can further evaluate the psychometric properties of the ToM task using the shared dataset; code for reproducing these reliability analyses is available on OSF (https://osf.io/g5zpv/).

### 3.6 Additional Resources

We provide a running list of open datasets that include ToM reasoning scores (measured using any task) in childhood (https://osf.io/g5zpv/).

## Limitations

To facilitate a naturalistic story-telling experience and promote children's engagement, experimenters administered the task from memory and without interruptions to reference a script. As such, one limitation entails missing datapoints and data acquired in a non-standardized way (e.g., questions asked in reverse order), due to experimenter error. These errors are indicated in the dataset (as are non-experimenter related errors, e.g., disruptions from parents). Another limitation is that coding was, for the most part, done by a single coder. Coders were trained by coding previously coded data and discussing questions and discrepancies with trained coders; this process continued until there were no questions/discrepancies. Multiple coders viewed a video recording to agree on coding when a coding decision was difficult or a free response was difficult to understand. Dataset curation involved checking for consistency in coding across coders and projects using the transcriptions (rather than by referring to videos). Instances of inconsistent coding were corrected (to be in line with the majority coding decision) such that a particular response was considered correct (or incorrect) consistently across coders and projects. Video data are not yet publicly released; individuals seeking access to video data should contact Drs. Hilary Richardson (hilary.richardson@ed.ac.uk) and Rebecca Saxe (saxe@mit.edu). Finally, we offer a cautionary note that creating criteria for coding free responses is challenging and coding free responses according to established criteria involves a level of subjective decision making.

## Ethics statement

Children signed an assent form and parents signed a consent form approved by the Committee on the Use of Humans as Experimental Subjects (COUHES) at MIT (protocols 0809002909, 0907003337, and 1502006952) and in accordance with the Declaration of Helsinki. Shared data have been fully anonymized.

## CRediT authorship contribution statement

**Koraima Sotomayor-Enriquez:** Data curation, Visualization, Writing – original draft, Writing – review & editing. **Hyowon Gweon:** Conceptualization, Methodology, Investigation, Writing – review & editing. **Rebecca Saxe:** Conceptualization, Methodology, Investigation, Resources, Writing – review & editing, Funding acquisition. **Hilary Richardson:** Conceptualization, Methodology, Investigation, Data curation, Writing – original draft, Writing – review & editing, Supervision.

## Data Availability

Theory of mind booklet task and open dataset (Original data) (Open Science Framework)
